# Fairness Creams in South Asia—A Case of Disease Mongering?

**DOI:** 10.1371/journal.pmed.0030315

**Published:** 2006-07-25

**Authors:** P. Ravi Shankar, Bishnu Rath Giri, Subish Palaian

**Affiliations:** **1**Manipal College of Medical SciencesPokharaNepal

We read with interest the article by Moynihan and Henry on disease mongering [ 1]. The authors argued that disease mongering is the opportunistic exploitation of a widespread anxiety about frailty and of faith in scientific advance and “innovation.”

In South Asia there is a widespread preference for “fair skin” and this has been exploited by the manufacturers of “fairness creams.” “White” skin has a colonial connotation of power and superiority. The emergence of a “paler” global entertainment industry has served as a fillip to the marketing of an international beauty ideal [ 2]. Beauty pageant winners in India are all extraordinarily tall and breathtakingly slim, have light honey-colored skin, and peddle Western ideals of beauty [ 3]. South Asian culture has carried within itself a capacity for female objectification. Matrimonial columns and Web sites reveal the influence of a young woman's skin color on her marketability to marriage partners [ 3].

The craze for modern fairness creams has emerged in the last fifty years [ 2]. International cosmetics giants were the initial manufacturers, but these days Indian and South Asian companies are playing an important role in the skin bleaching and cosmetic markets [ 2, 3]. Fairness creams have been estimated to account for up to 40% of the profits of the cosmetics industry [ 3]. Recently, a fairness cream has been launched exclusively for men [ 4].

Advertisements aim to produce a hierarchy of values based on the notion that “fairness” is an object of desire [ 2]. Being fair has been represented as an active process. Regular use of fairness creams has been claimed to halt the production of melanin and to bring out “natural” beauty.

Promoting a particular body image or behavior pattern as the preferred one and then selling medicines or products to help people attain the particular ideal may be regarded as disease mongering [ 5]. Fairness cream manufacturers have exploited the preference for fair skin, portrayed it as a necessary prerequisite for success, and promoted the use of their product to achieve the ideal. Controlled studies on the efficacy and safety of fairness creams are lacking.

Disease mongering companies form alliances with doctors, consumer groups, and the media to promote sales of their drugs. Fairness cream manufacturers sponsor beauty pageants and carry out an advertising blitz in the print and audiovisual media [ 3]. They create hype about their product. Many leading manufacturers have expanded their range to include lotions, cold creams, and soaps.

Most fairness creams are nonprescription products, and the medical profession may not be the main target of marketing professionals. However, doctors as responsible and respected members of society have an important role to play in spreading awareness about this racial distortion of body image. Fairness creams may satisfy many of the criteria of disease mongering. The issues of freedom of choice, economic impact (personal and on the society), profits, social issues, and ideal body image should be seriously debated.
